# ALK Knock-In Reporter Reveals APE1 as a Negative Regulator of *EML4-ALK* Formation

**DOI:** 10.3390/ijms27135676

**Published:** 2026-06-24

**Authors:** Matvey M. Murashko, Ekaterina M. Stasevich, Kirill V. Korneev, Anna D. Dorfman, Denis E. Demin, Elvina A. Prikhodko, Elina A. Zheremyan, Aksinya N. Uvarova, Anton M. Schwartz, Dmitry V. Kuprash

**Affiliations:** 1 Center for Precision Genetic Technologies for Medicine, Engelhardt Institute of Molecular Biology, Russian Academy of Sciences, 32 Vavilova Street, Moscow 119991, Russia; mmmurashko@gmail.com (M.M.M.);; 2 Engelhardt Institute of Molecular Biology, Russian Academy of Sciences, 32 Vavilova Street, Moscow 119991, Russia; 3 Department of Human Biology, Faculty of Natural Sciences, University of Haifa, 199 Abba Khoushy Avenue, Mount Carmel, Haifa 3498838, Israel; aschwart2@univ.haifa.ac.il

**Keywords:** *EML4-ALK*, *ALK* rearrangement, non-small cell lung cancer, oncogenic gene fusion, chromosomal rearrangement, *APEX1*/APE1, DNA repair, CRISPR-Cas9, fluorescent reporter

## Abstract

Chromosomal rearrangements that lead to the formation of oncogenic gene fusions, such as *EML4-ALK*, are thought to arise from incorrect repair of double-strand breaks in DNA. However, the mechanisms and factors driving rearrangement formation remain poorly understood, and analysis of these processes is limited by detection methods that are labor-intensive, low-throughput, and not readily quantitative at single-cell resolution. Here, we developed a genetically encoded ALK reporter based on A549 lung adenocarcinoma cells, created by inserting an *ALK*-P2A-mCherry cassette into the endogenous *ALK* locus, so that induced *EML4-ALK* fusion activated mCherry fluorescence. Reporter activation yielded a readily quantifiable mCherry-positive subpopulation that could be measured and enriched by flow cytometry and correlated with *EML4-ALK* levels. Using this platform, we combined CRISPR-mediated rearrangement induction with knockdown of DNA repair factors using RNA interference. Of the factors involved in base excision repair, homologous recombination-related pathways and canonical non-homologous end joining, knockdown of the *APEX1* gene encoding apurinic endonuclease 1 (APE1) selectively increased *EML4-ALK* levels both in the reporter cell line and in parental A549 cells. Together, this work provides a sensitive, single-cell A549-based ALK reporter platform and a framework for future studies aimed at identifying cellular and environmental factors that modulate oncogenic *EML4-ALK* rearrangement formation.

## 1. Introduction

Genomic instability is a hallmark of cancer and often drives tumor progression through different classes of DNA alterations [1]. Among these, chromosomal rearrangements are one of the major sources of oncogenic drivers [2]. Translocations, inversions, duplications, or deletions frequently lead to gene fusions that encode constitutively active proteins, deregulate gene expression, and activate downstream pathways [3,4]. Classic examples include *BCR-ABL1* in chronic myeloid leukemia, *RET-PTC* in papillary thyroid carcinoma, *TMPRSS2-ERG* in prostate cancer, and *EML4-ALK* in lung cancer. These fusions not only define tumor subtypes but also provide diagnostic markers and therapeutic targets [5,6].

Among oncogenic fusions, rearrangements involving the anaplastic lymphoma kinase (*ALK*) gene are one of the most intensively studied. ALK is a receptor tyrosine kinase of the insulin receptor superfamily encoded on chromosome 2p23 and normally expressed at low levels in the nervous system and select peripheral tissues. Upon ligand-induced dimerization or autophosphorylation, ALK activates downstream signaling cascades, including JAK-STAT, PI3K-AKT-mTOR, and RAS-MAPK, that regulate cell proliferation, survival and differentiation [7,8]. Oncogenic *ALK* rearrangements are best characterized in non-small cell lung cancers (NSCLC) where they occur in approximately 3–7% of cases, enriched in younger patients with adenocarcinoma histology and in never- or light-smokers. *ALK* fusions also act as key drivers in other malignancies such as anaplastic large cell lymphoma (ALCL), inflammatory myofibroblastic tumors (IMT), neuroblastoma, and subsets of thyroid and other solid cancers [9,10].

Although they arise in different tissues and tumor types, oncogenic *ALK* rearrangements share a common structure: the 3′-kinase-encoding region of *ALK* fuses to various 5′-partner genes that promote constitutive activation of ALK. In NSCLC, inv(2)(p21p23) generates *EML4-ALK*, which accounts for the majority of *ALK* rearrangements, whereas a broader repertoire of partners (such as *KIF5B*, *STRN*, *NPM1*, *TPM3/4*, *CLTC*, *RANBP2*, and others) is less common and is more frequently reported in other tumor types [9,11]. Moreover, multiple *EML4-ALK* variants exist, with variants 1 (E13; A20) and 3 (E6a/b; A20) being the most common [12]. The common 3′-kinase-encoding part of ALK kinase underlies the response of cancers with different *ALK* rearrangements to ALK inhibition, regardless of tumor type [7].

Although the clinical and molecular aspects of chromosomal rearrangements are well characterized across many tumor types, the question of how oncogenic rearrangements arise in somatic cells remains unsolved. Chromosomal translocations and other structural variants are thought to arise primarily from misrepaired DNA double-strand breaks (DSBs), and deregulation of DNA repair networks has been consistently linked to characteristic patterns of structural variation and increased chromosomal rearrangements [13,14,15,16]. Proposed mechanisms include a combination of DNA recombination, repair, and replication-based mechanisms, including error-prone DNA repair of double-strand breaks and replication fork stalling or collapse, often in the context of repetitive elements and higher-order genome architecture that bring susceptible loci into proximity [17,18].

This process can also be viewed in the context of clastogenesis, where DNA strand breaks induced by physical or chemical agents generate chromosome fragments that may be incorrectly rejoined, inserted into other genomic regions, or incorporated into translocations [19]. In addition to tobacco smoke, occupational or environmental exposure to genotoxicants such as benzene, polycyclic aromatic hydrocarbons, pesticides, antineoplastic drugs, and ionizing radiation may contribute to DNA damage, chromosomal aberrations, and genomic instability, although *ALK*-positive NSCLCs frequently occur in never-smokers [10,20,21,22,23,24,25].

Emerging works also mention RNA molecules, including long non-coding RNAs (lncRNAs), as active participants in rearrangement formation: synthetic fusion RNAs and mRNA have been shown to stimulate specific DNA fusions [26,27,28]. Recently, we showed that the *CACSTL1* lncRNA facilitated *CCDC6-RET* inversion in thyroid cells via complementarity to introns of fused genes, providing direct evidence that lncRNAs can stimulate driver rearrangements [26].

Experimental analysis of oncogenic chromosomal rearrangements has been further advanced by genome-editing approaches that induce defined DNA double-strand breaks, in particular, dual-guide CRISPR-Cas9 systems have enabled modeling of deletions, inversions, and translocations in cultured cells and animal models [29,30]. However, detection of chromosomal rearrangements remains time-consuming and technically challenging, especially in settings that require systematic screening or dynamic quantification of rearrangement formation. Usually, gene fusions are identified using one of the following techniques: fluorescence in situ hybridization (FISH), immunohistochemistry, reverse transcription quantitative PCR (RT-qPCR) or DNA/RNA sequencing. Each has limitations such as labor-intensive approaches, dependence on nucleic acid quality, restricted coverage of breakpoint diversity, and limited suitability for high-throughput functional screens [31,32,33]. Fluorescent reporters partly overcome this limitation by enabling flow-cytometric detection of induced rearrangements. However, many such systems use artificial reporter configurations or measure DSB-repair activity rather than formation of oncogenic chromosomal rearrangements in their native genomic configuration [34,35,36].

To overcome these constraints, we developed a genetically encoded A549-based ALK reporter in which chromosomal inversion activates expression of a fluorescent protein, enabling rapid, sensitive and quantitative detection of chromosomal rearrangements at single-cell resolution by flow cytometry. Moreover, in the present study, we used this reporter together with RNA interference-mediated knockdown of genes involved in key DNA repair pathways to analyze how it affects the efficiency of *EML4-ALK* formation. Although *EML4-ALK* was used here as a model rearrangement in A549 lung adenocarcinoma cells, this strategy may be adaptable to other cellular models and other defined chromosomal rearrangements. However, such applications will require separate validation.

## 2. Results

### 2.1. EML4-ALK Fluorescent Reporter in A549 Cells

**Figure 1:**
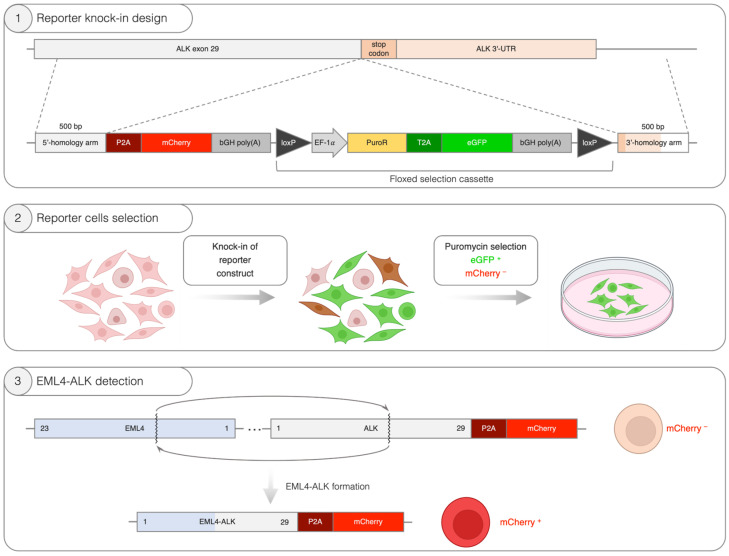
Design, selection, and functional principle of the ALK reporter A549 cell line for detection of endogenous *EML4-ALK* formation. (**1**) Schematic of the reporter knock-in design at the endogenous *ALK* locus. A P2A-mCherry reporter together with an EF1α-PuroR-T2A-eGFP selection cassette was inserted immediately upstream of the *ALK* stop codon by homology-directed repair (HDR) using 5′ and 3′ homology arms. (**2**) Selection of ALK reporter A549 cell line. Following knock-in of the reporter construct, cells were enriched by puromycin selection for 2 weeks and eGFP-positive, mCherry-negative sorting. (**3**) Principle of reporter activation. In the native state, the endogenous *ALK* locus remains silent in A549 cells and reporter cells are mCherry-negative. CRISPR-induced formation of the *EML4-ALK* fusion increases expression of the 3′-region of the *ALK* gene, leading to co-expression of mCherry and allowing detection of fusion-positive cells at the single-cell level using flow cytometry.

To enable rapid, single-cell quantification of endogenous *EML4-ALK* rearrangements, ALK reporter A549 cell line was designed by knocking in a P2A-mCherry together with an EF1α-PuroR-T2A-eGFP selection cassette before the endogenous stop codon of *ALK* (Figure 1). Knock-in and Puromycin selection were performed in four independent biological repeats. The percent of selected GFP-positive and mCherry-negative cells post Puromycin selection was in a range from 44 to 70% (Figure S1). The correct insert was confirmed by Sanger sequencing.

**Figure 2:**
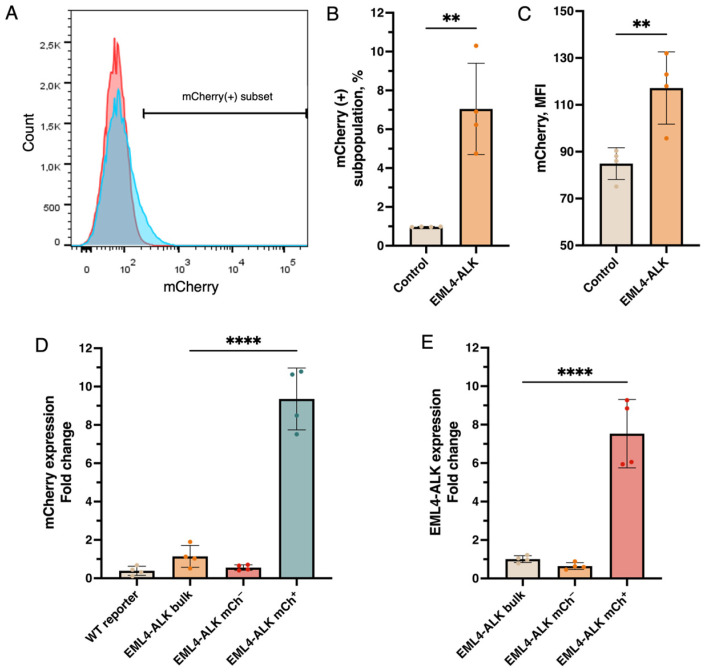
Validation of an ALK reporter A549 cell line for single-cell detection of CRISPR-induced *EML4-ALK* rearrangements in A549 cells. (**A**) Representative flow-cytometry profiles showing mCherry fluorescence in reporter cells with or without *EML4-ALK* induction. (**B**) Percentage of cells within the mCherry^+^ subset in non-induced control reporter cells and in reporter cells after *EML4-ALK* induction. The non-induced control refers to ALK reporter A549 cells processed in parallel but not transfected with the *EML4-ALK*-inducing CRISPR-Cas9 constructs. An unpaired *t*-test was used. (**C**) Quantification of mCherry mean fluorescence intensity (MFI) in non-induced control reporter cells and reporter cells after *EML4-ALK* induction. An unpaired *t*-test was used. (**D**) RT-qPCR quantification of mCherry transcript levels in A549 reporter cells across the indicated conditions: wild-type (WT) reporter, *EML4-ALK* bulk (*EML4-ALK* induced without sorting), *EML4-ALK* mCherry^−^, and *EML4-ALK* mCherry^+^ subsets isolated after induction. Expression is shown as fold change relative to *EML4-ALK* bulk, normalized to β-actin and calculated using the 2^−ΔΔCt^ method. One-way ANOVA was used. (**E**) RT-qPCR quantification of the *EML4-ALK* fusion junction transcript in *EML4-ALK* bulk, *EML4-ALK* mCherry^−^, and *EML4-ALK* mCherry^+^ populations isolated after induction. Relative expression was normalized to *β-actin* and calculated using the 2^−ΔΔCt^ method. *EML4-ALK* junction site was verified with Sanger sequencing. One-way ANOVA was used. Data are presented as mean ± SD. Dots represent individual biological replicates. N = 4 biological replicates are shown. **—*p* < 0.005; ****—*p* < 0.0001.

To check whether chromosomal inversion causes mCherry activation, *EML4-ALK* was induced in each pool of selected reporter cells. CRISPR-Cas9 transfection with guide RNAs (gRNAs) targeting introns of *EML4* and *ALK* resulted in a reproducible rightward shift in mCherry fluorescence in all biological repeats. *EML4-ALK* induction did not generate a fully distinct mCherry-positive population, likely because activation of a single endogenous mCherry reporter allele produces a relatively low fluorescence signal that partially overlaps with background fluorescence. Reporter activation was quantified as the proportion of cells in the mCherry^+^ subset. To provide an empirical fluorescence standard for this readout, the mCherry^+^ threshold was set at the 99th percentile of non-induced reporter cells, corresponding to approximately 1% background positivity, and the same fixed gate was applied to induced samples. Thus, the percentages shown represent the fraction of analyzed singlet events above this fixed mCherry threshold gate. Induction of *EML4-ALK* showed a clear increase in the mCherry^+^ subset compared to control samples (Figure 2A,B and Figure S2). In addition, quantification of mCherry mean fluorescence intensity (MFI) showed a reproducible increase after *EML4-ALK* induction (Figure 2C), further supporting reporter activation at the population level.

To further confirm that the mCherry signal reflects *EML4-ALK* formation, reporter cells after *EML4-ALK* induction were separated by flow cytometry into mCherry-low and mCherry-high subpopulations for further quantification of both mCherry and *EML4-ALK* (E13; A20) transcripts by RT-qPCR. The mCherry-high fraction showed strong enrichment of mCherry (Figure 2D) and, importantly, a concordant enrichment of *EML4-ALK* fusion transcript (Figure 2E) relative to the unsorted bulk and the mCherry-low populations, indicating that mCherry activation depicts *EML4-ALK* formation rather than nonspecific changes in fluorescence.

### 2.2. Quantification of EML4-ALK in ALK Reporter Under DNA Repair Factor Knockdown

**Figure 3:**
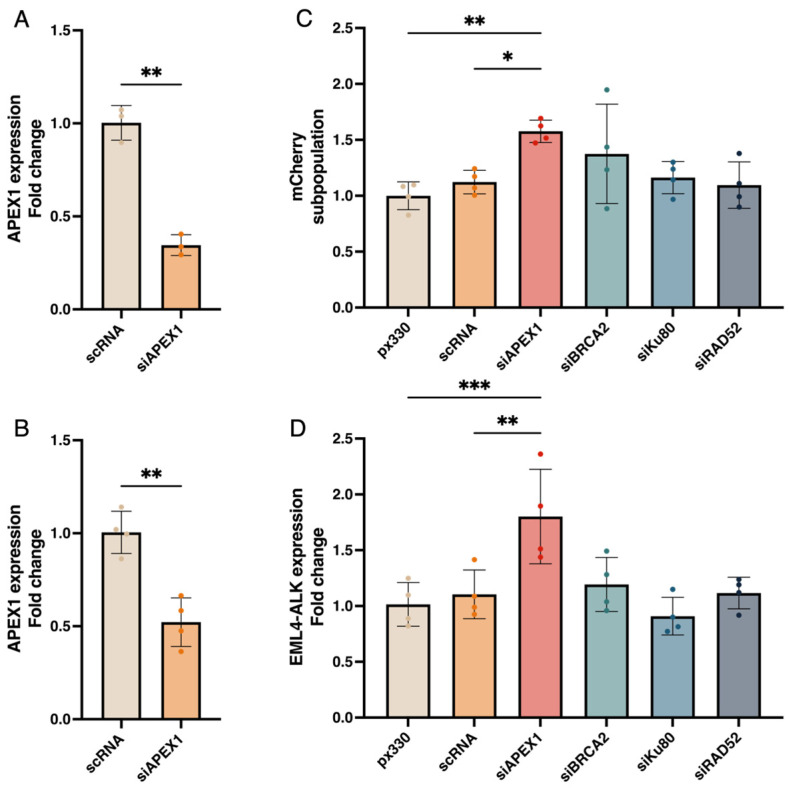
Effect of DNA repair factor knockdown on CRISPR-induced *EML4-ALK* rearrangement formation in ALK reporter A549 cells. (**A**,**B**) Knockdown efficiency of *APEX1* mRNA in the ALK reporter A549 cell line was measured 48 h (**A**) and 7 days (**B**) after transfection. Transcript levels were quantified relative to cells treated with non-targeting siRNA and normalized to β-actin using the 2^−ΔΔCt^ method. (**C**) Fraction of mCherry-positive cells measured by flow cytometry 1 week after co-delivery of siRNAs and CRISPR-Cas9 constructs inducing *EML4-ALK*, normalized to the *EML4-ALK* induction without siRNA (px330). *APEX1* knockdown increased reporter activation compared with control conditions, whereas depletion of *BRCA2*, *XRCC5*, or *RAD52* did not significantly alter the mCherry-positive fraction. (**D**) RT-qPCR quantification of the *EML4-ALK* fusion-junction transcript under the same conditions. Expression was normalized to β-actin and calculated by the 2^−ΔΔCt^ method. *APEX1* knockdown increased *EML4-ALK* junction levels, while knockdown of *BRCA2*, *XRCC5*, or *RAD52* had no significant effect. For panels (**A**,**B**), an unpaired *t*-test was used. For panels (**C**,**D**), one-way ANOVA was used. The graph represents mean values ± SD from N = 3 for panel A and N = 4 biological replicates for panels B, C and D. Dots represent individual biological replicates. *—*p* < 0.05; **—*p* < 0.005; ***—*p* < 0.0005.

We next studied how depletion of DNA repair factors affects *EML4-ALK* formation and used this experiment as a proof-of-principle application of the ALK reporter system. Small interfering RNA (siRNA) knockdown of selected genes involved in DNA repair was performed in the ALK reporter A549 cell line. *APEX1* (APE1), involved in base excision repair, *BRCA2* and *RAD52*, involved in homologous recombination-related repair, and *XRCC5* (Ku80), as a representative factor of canonical non-homologous end joining (canonical NHEJ, C-NHEJ), were targeted, and knockdown efficiency was assessed at the mRNA level by RT-qPCR (Figure 3A,B and Figure S3). The siRNAs used in this study were selected based on previously published RNA interference (RNAi) studies targeting the corresponding DNA repair factors. In particular, the *APEX1* siRNA was chosen based on previous studies in A549 and other non-small cell lung cancer cell lines, where the same sequence was associated with reduced APE1 protein levels by Western blot [37,38].

*EML4-ALK* was induced using the same CRISPR-Cas9 approach as above, with siRNAs and CRISPR-Cas9 constructs delivered together. Following the rearrangement induction protocol, the fraction of mCherry-positive cells was measured by flow cytometry one week after transfection. Under these conditions, *APEX1* knockdown increased the level of *EML4-ALK* as compared to control conditions (px330 without siRNA and px330 with non-targeting scrambled RNA (scRNA)), whereas depletion of *BRCA2*, *XRCC5*, or *RAD52* did not result in a significant change in reporter activation (Figure 3C). The same pattern was observed by RT-qPCR of the *EML4-ALK* junction (Figure 3D).

Together, these results demonstrate that the reporter provides a convenient assay to quantify *EML4-ALK* formation at single-cell resolution and can be applied to study factors affecting chromosomal rearrangement formation.

### 2.3. Reporter-Independent Assessment of Effects on EML4-ALK Formation in Parental A549 Cells

**Figure 4:**
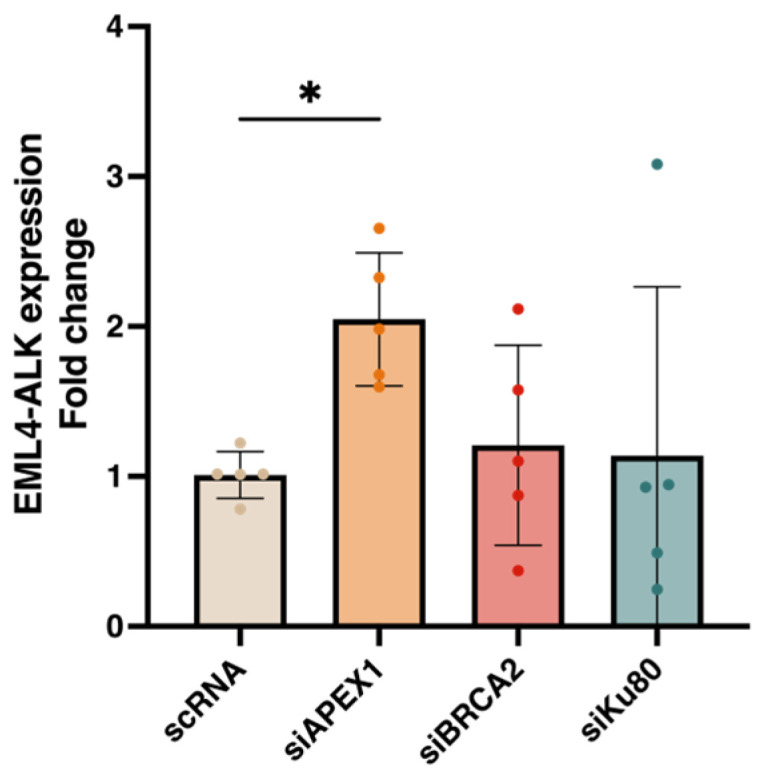
Reporter-independent assessment of *APEX1* knockdown effect in parental A549 WT cells. RT-qPCR quantification of the *EML4-ALK* fusion junction in A549 WT cells 7 days after transfection of *EML4-ALK*-inducing CRISPR-Cas9 constructs with scRNA, siAPEX1, siBRCA2, or siRAD52. Expression was normalized to β-actin and calculated using the 2^−ΔΔCt^ method relative to the scRNA control. One-way ANOVA was used. The graph represents mean values ± SD from N = 5 biological replicates. Dots represent individual biological replicates. *—*p* < 0.05.

To test whether the effect observed in the reporter system was independent of the knock-in design or fluorescence-based detection, we next tested a reduced siRNA panel in the parental A549 cells and quantified *EML4-ALK* formation directly by RT-qPCR. Based on the reporter-screening results, *APEX1* was selected as the candidate of interest, while two additional siRNAs that did not show a significant effect in the reporter assay were included as negative controls together with a non-targeting scRNA. siRNAs were co-delivered with the CRISPR-Cas9 constructs used to induce *EML4-ALK*, and samples were collected 7 days later for junction RT-qPCR analysis. Under these conditions, *APEX1* knockdown increased the *EML4-ALK* signal relative to the non-targeting siRNA control, whereas the other tested knockdowns did not cause significant changes (Figure 4). Thus, the increase in *EML4-ALK* formation upon *APEX1* depletion was reproduced in the parental cell line, indicating that this effect was not dependent on the reporter construct or fluorescence-based readout.

## 3. Discussion

Our data demonstrated that knock-in of the *ALK*-P2A-mCherry reporter in A549 cells allowed a direct fluorescent detection of endogenous *ALK* rearrangement and was compatible with flow-cytometric quantification and sorting. This opens up new opportunities for studying the biology of chromosomal rearrangement formation in settings where traditional identification of structural variants remains time-consuming. However, the partial overlap between induced and control mCherry fluorescence distributions suggests that future reporter designs aimed at detecting rare rearrangement events could benefit from substituting mCherry with EGFP, since EGFP is substantially brighter than mCherry, facilitating more robust detection and sorting of low-positive subpopulations [39].

The present study focused on the development and validation of the reporter in A549 lung adenocarcinoma cells. Because reporter characteristics and efficiency may vary with cell-type-specific *ALK* expression, chromatin accessibility, DNA repair activity, and transfection efficiency, application of this approach to other tumor types or rearrangements will require independent validation.

The ability of the reporter to detect *ALK* fusions other than *EML4-ALK* also remains to be determined. Because the reporter cassette is inserted at the 3′ end of the endogenous *ALK* gene, rearrangements involving other 5′ partners may lead to mCherry activation if they induce transcription of the reporter-tagged 3′ ALK region. However, this possibility was not directly tested in the present study and will require experimental validation.

Another interesting observation was the increase in *EML4-ALK* formation upon *APEX1* depletion. APE1 is traditionally considered a base excision repair (BER) factor that protects the genome, and its nuclease activity can generate intermediate single-strand breaks during the processing of abasic sites [40]. This mechanism may also be relevant because two single-strand repair intermediates located on opposite DNA strands and close to each other can result in double-strand breaks. A similar principle operates during immunoglobulin class switch recombination, where APE1/APE2-dependent processing of abasic sites contributes to S-region DNA breaks that are subsequently repaired by C-NHEJ or alternative end joining (A-EJ) [41]. However, some evidence suggests that the loss of APE1 may affect DNA repair in a way that promotes genomic rearrangements. In multiple myeloma models, inhibition of *APEX1/APEX2* reduced homologous recombination activity through suppression of RAD51, interaction with key homologous recombination (HR) regulators, and p73-dependent control of RAD51 expression [42]. Because HR disruption can increase dependency on NHEJ (including alternative NHEJ), which is more prone to mis-repair, this could mechanistically favor chromosomal rearrangements such as *EML4-ALK* [13]. In this context, the *XRCC5*/Ku80 result provides only a partial assessment of C-NHEJ involvement. Although Ku80 was included as a representative NHEJ factor, *XRCC5* knockdown alone does not exclude the involvement of C-NHEJ in *EML4-ALK* formation. Future studies targeting downstream ligation factors such as XRCC4 and DNA ligase IV (LIG4) will be useful to clarify the contribution of this pathway.

A broader role for APE1 beyond BER has also been demonstrated in recent studies linking APE1 to DSB repair pathway selection and timing after oxidative stress. APE1 inhibition disrupted timely DSB repair and led to DSB accumulation accompanied by increased ATM activation [43]. This delayed clearance of breaks may prolong coexistence of multiple DNA ends, thereby increasing the probability of aberrant joining events. In addition, APE1 has been proposed to act as a regulator of ATR- and ATM-mediated DNA damage response (DDR) signaling [44]. Moreover, siRNA-mediated APEX1 depletion or APE1 inhibitors impair oxidative stress-induced ATR DDR signaling [45]. Together, these published data support the idea that APE1 reshapes damage processing and checkpoint signaling. In our system, reduced HR and altered DDR kinetics can possibly explain increased *EML4-ALK* formation during *APEX1* knockdown.

In addition, APE1 is involved in other *ALK*-fusion-driven cancers. APE1/REF1 was reported to stabilize *NPM1-ALK* fusion protein oligomers and reduce sensitivity to ALK tyrosine kinase inhibitors (TKIs), while disrupting this interaction increased the efficacy of crizotinib and alectinib [46]. Although this involves a different *ALK* fusion, it supports the idea that APE1 can affect *ALK*-driven oncogenic states at multiple levels (DNA repair/DDR and fusion protein biology).

Given the siRNA-based design of these experiments, further validation using additional approaches will be useful to define the role of *APEX1* in *EML4-ALK* formation more precisely. The parental A549 experiment indicates that the observed increase in *EML4-ALK* is not dependent on the reporter construct or fluorescence-based detection. The siRNAs used in this work were selected based on published RNAi studies, and the *APEX1* siRNA has previously been tested in A549 and other non-small cell lung cancer models with Western blot confirmation of reduced APE1 protein levels [37,38]. Future studies using APE1 inhibitors, additional knockdown strategies, genetic knockout, and rescue experiments will help clarify the mechanisms by which APE1 may influence *EML4-ALK* formation, including possible effects on DNA repair pathway choice, DNA damage response signaling, or accumulation of repair intermediates.

Although the present study used CRISPR-Cas9-induced DNA breaks to model *EML4-ALK* formation, environmental and occupational genotoxicants may also promote chromosomal rearrangements by increasing DNA damage and chromosomal instability [20,21,22,23,24,25]. Direct evidence linking such exposures to *EML4-ALK* formation remains limited, but the reporter system developed here may provide a useful platform for testing whether defined genotoxic stresses modulate oncogenic rearrangement formation.

Finally, returning to the reporter line, the present targeted siRNA experiment demonstrates a proof-of-principle application of the system for testing candidate factors that influence *ALK* rearrangement formation. Fluorescent signal detection enables direct quantification and enrichment of rearranged cells, suggesting that, after further validation, the reporter may be compatible with pooled CRISPRi/CRISPRa, open reading frame (ORF)/lncRNA expression libraries, and other massively parallel screening formats. Such future applications could provide a systematic approach to investigating thousands of coding and non-coding candidates, including pathways that are difficult to capture in targeted studies given the remaining gaps in our understanding of chromosomal rearrangement formation.

## 4. Materials and Methods

### 4.1. Cell Culture

A549 cell line (ATCC CCL-185), kindly provided by Prof. Dr. Vladimir Prassolov, was cultured in Cell Culture Treated Flasks at 37 °C in the presence of 5% CO_2_ in DMEM (glucose 4.5 g/L, with HEPES and stabilized L-glutamine) (BioinnLabs, Rostov-on-Don, Russia, cat. no. bn-3A2A) supplemented with 10% fetal bovine serum (Corning, Corning, NY, USA, cat. no. 35-079-CV), 100 U ml^−1^ penicillin and 100 µg ml^−1^ streptomycin (Paneco, Moscow, Russia, cat. no. A074), and 1x MEM Non-Essential Amino Acids (Paneco, Moscow, Russia, cat. no. 114/50). All experiments were performed with mycoplasma-free cells. The cell line was authenticated using STR profiling.

### 4.2. Construction of the HDR Donor Template

An HDR (homology-directed repair) donor construct was designed to target the last exon of *ALK* upstream of the stop codon. A 500-bp 5′ homology arm (−500 to −1 bp relative to the stop codon) and a 500-bp 3′ homology arm spanning the stop-codon region and downstream sequence were amplified from genomic DNA of the A549 cell line. Guide RNA spanning the stop codon/3′ untranslated region junction was selected, with the corresponding protospacer-adjacent motif (PAM) partially located within the last coding codon of exon 29 (Figure S4). A silent mutation was introduced in the last coding codon, changing CCC to CCT without altering the encoded proline, as an additional barrier. The knock-in cassette contained a P2A peptide fused in-frame to mCherry (co-expressed with ALK via a P2A peptide), followed by a bovine growth hormone polyadenylation signal (bGH poly(A)), and a PuroR—T2A—eGFP cassette under the EF1α promoter and terminated with a second bGH poly(A) signal flanked by loxP sites for possible Cre-mediated removal (Figure S5).

The pGL3 plasmid backbone was used for assembly, where *firefly luciferase* gene was removed and substituted with the HDR donor construct. All elements were assembled in the following order: 5′ homology arm—P2A—mCherry—bGH poly(A)—loxP—EF1α—PuroR—T2A—eGFP—bGH poly(A)—loxP—3′ homology arm. For cloning, fragments were digested with combinations of Type II and Type IIS restriction enzymes and ligated into the linearized backbone. Where restriction sites were not available, PCR overlap extension was used to generate contiguous fragments before ligation. The correctness of the sequence and junctions was verified using colony PCR and Sanger sequencing.

Cloning was performed using Tersus polymerase (Evrogen, Moscow, Russia, cat. no. PK123S) and restriction enzymes (Thermo Fisher Scientific, Waltham, MA, USA).

### 4.3. Knock-In of Reporter Construct

Guide RNAs (gRNAs) for SpCas9 targeting the *ALK* stop codon region were designed using ChopChop (chopchop.cbu.uib.no (accessed on 21 June 2026)) [47]. Candidate gRNAs were cloned into the pSpCas9(BB)-2A-GFP (px458) construct, which was a gift from Feng Zhang (Addgene plasmid #48138, Watertown, MA, USA) [48], and the gRNA spanning the stop codon with its PAM sequence covering the last coding codon of exon 29 of *ALK* was selected for knock-in experiments (5′-GTGAGTGTGCGACCGAGCTC-3′). 1 × 10^6^ of A549 cells were transfected with 5 μg of HDR donor template plasmid and 1 μg of px458-gRNA construct.

### 4.4. Transfection

Transfection of A549 cells was performed using electroporation with the Neon Transfection System (Life Technologies, Carlsbad, CA, USA) by two 30 ms pulses at 1230 V in 100 μL tips designed for this instrument. Cells were transferred to a 6-well plate, 2 mL of complete growth medium per well.

### 4.5. Selection of Edited Cells

After 24 h, cells were sorted by GFP fluorescence using fluorescence-activated cell sorting (FACS) on the FACSAria™ III Cell Sorter (BD Biosciences, San Jose, CA, USA). Puromycin (Sigma-Aldrich, St. Louis, MO, USA, cat. no. P7255) was added 24 h after sorting at a final concentration of 10 µg mL^−1^, and resistant cells were maintained under selection for 2 weeks prior to downstream analyses. After 2 weeks, additional sorting was performed, GFP-positive and mCherry-negative cells were selected.

### 4.6. Induction of Chromosomal Rearrangements

*EML4-ALK* rearrangement was induced with CRISPR-Cas9, as described in the previous article [26]. Guide RNAs targeting intron 13 of the *EML4* gene (5′-GTCCTACAGAATTAGTTACC-3′) and an intron 20 of the *ALK* gene (5′-GGACCGACCGTGATCAGATT-3′) were designed with ChopChop (chopchop.cbu.uib.no (accessed on 21 June 2026)) and cloned into the pU6-(BbsI)_CBh-Cas9-T2A-BFP (px330) construct which was a gift from Ralf Kuehn (Addgene plasmid # 64323; Watertown, MA, USA) for experiments with ALK reporter A549 cell line and cloned into the pSpCas9(BB)-2A-GFP (px458) construct which was a gift from Feng Zhang (Addgene plasmid #48138, Watertown, MA, USA) for experiments with A549 WT [48,49]. For induction, 1 × 10^6^ cells (ALK reporter A549 or A549 WT) were co-transfected with two CRISPR-Cas9 plasmids, 5 μg each, targeting two introns. The cells were placed in a 6-well plate in 2 mL of complete medium.

At 24 h post-transfection, the medium was replaced with fresh complete medium. The cells were then maintained under standard culture conditions for 7 days. This time point was selected to allow sufficient time for CRISPR-Cas9 expression, DNA cleavage, chromosomal inversion formation, and subsequent accumulation and maturation of the mCherry reporter signal. By the end of this period, cells typically reached 80% to 90% confluence, providing sufficient material for downstream analysis of *EML4-ALK* fusion formation by RT-qPCR and flow cytometry.

### 4.7. siRNA Knockdown

For siRNA-mediated knockdown, cells were co-electroporated with CRISPR-Cas9 plasmid used for *EML4-ALK* rearrangement induction and siRNA in a single Neon electroporation reaction. 1 μL of siRNA of 100 μM stock solution was added to the electroporation mixture together with the CRISPR-Cas9 plasmid immediately before electroporation. Electroporation was performed under the same conditions as described above for plasmid delivery, after which cells were transferred to complete growth medium. The following siRNAs were used (sense strand, 5′-3′): *APEX1* (APE1)—GUCUGGUACGACUGGAGUACC, *BRCA2*—AACAACAAUUACGAACCAAAC, *XRCC5* (Ku80)—AAGCAUAACUAUGAGUGUUUA, *RAD52*—GGAUGGUUCAUAUCAUGAA, scrambled RNA (scRNA)—GGCUUAAACGAAUGAUAAU. All siRNAs were chemically synthesized by DNA-Synthesis (Moscow, Russia) based on the indicated published sequences.

### 4.8. Flow Cytometry and Fluorescence-Activated Cell Sorting

Cells were analyzed and sorted on a BD FACSAria III cell sorter (BD Biosciences, San Jose, CA, USA) using a standard gating strategy. Briefly, A549 cells were first identified by FSC-A/SSC-A gating, followed by singlet discrimination using FSC-A/FSC-H. Depending on the experimental design, GFP and/or mCherry fluorescence was then assessed. During reporter cell line selection, A549 WT cells were used to assess background fluorescence and set the GFP gate. In experiments using the reporter cell line, control reporter cells were used to set the threshold mCherry signal and define the mCherry^+^ subset. Fluorescence-positive cells were quantified by flow cytometry and, when required, isolated by cell sorting. For each sample, at least 50,000 singlet events were acquired and used for downstream fluorescence analysis. Data were analyzed using FlowJo v10 Software (BD Biosciences, San Jose, CA, USA).

### 4.9. Flow-Cytometric Quantification of mCherry Reporter Activation

Because induction of *EML4-ALK* in the *ALK* reporter A549 cell line resulted in a rightward shift of the overall mCherry fluorescence distribution, rather than the appearance of a clearly separated mCherry-positive population, likely due to a relatively low brightness of mCherry at this expression level, reporter activation was quantified using two complementary readouts: the percentage of cells above a fixed mCherry threshold gate (“mCherry^+^ subset”) and the mCherry mean fluorescence intensity (MFI) of the analyzed singlet population. This fixed threshold gate was set using non-induced A549 reporter cells at the 99th percentile of the mCherry fluorescence distribution, so that approximately 1% of control cells fell within the gate. The same gate was then applied unchanged to induced samples, and the percentage of cells within the mCherry^+^ subset was used to quantify reporter activation.

### 4.10. RNA Isolation and cDNA Synthesis

Total RNA was isolated from 0.5–2 × 10^6^ cells using the ExtractRNA kit (Evrogen, Moscow, Russia, cat. no. BC032). To remove genomic DNA contamination, RNA was treated with DNase I (Biolabmix, Novosibirsk, Russia, cat. no. EM-100) and the enzyme was inactivated according to the manufacturer’s instructions. Reverse transcription was done with the MMLV RT kit (Evrogen, Moscow, Russia, cat. no. SK021) according to the supplier’s protocol. For complementary DNA (cDNA) synthesis, 2 µg of total RNA were used in a 20 µL reaction containing random hexamer primers and oligo(dT)_15_ in equal proportions.

### 4.11. Quantitative PCR

RT-qPCR was used to measure the expression of the chimeric transcript *EML4-ALK*, mCherry, and selected DNA repair genes. Reactions were prepared using qPCRmix-HS SYBR (Evrogen, Moscow, Russia, cat. no. PK147L) according to the manufacturer’s instructions. Each 25 μL reaction contained 1 μL of cDNA and primers at a final concentration of 200 nM each. Amplification was performed in technical duplicates for each sample and the mean cycle threshold (Ct) value was used for downstream analysis. Expression levels were normalized to *ACTB* (β-actin) as the internal reference gene, and relative expression was determined using the 2^−ΔΔCt^ method. Primers are provided in Table S1. Primer efficiencies were validated using the standard curve method and were greater than 90%.

### 4.12. DNA Sequencing

Correct knock-in of the reporter construct in A549 cells and the RT-qPCR amplicon spanning the *EML4-ALK* junction were verified by Sanger sequencing. Sequencing was performed at the EIMB RAS “Genome” sequencing center (Moscow, Russia).

### 4.13. Statistical Analysis

Statistical analysis was performed using GraphPad Prism, version 10 (GraphPad Software Inc., San Diego, CA, USA). Data are shown as mean ± SD. Two-group comparisons were performed using an unpaired Student’s *t*-test. Multiple-group comparisons were performed using one-way ANOVA with Tukey’s post hoc test. Differences were considered statistically significant at *p* < 0.05. Detailed information on sample sizes and statistical analyses for individual experiments is provided in the corresponding figure legends.

## 5. Conclusions

In this study, we developed an endogenous ALK knock-in reporter that enables flow-cytometric detection, quantification, and enrichment of cells with CRISPR-induced *EML4-ALK* rearrangement. The siRNA experiments with DNA repair factors, including *APEX1*, demonstrate a proof-of-principle application of this reporter for studying modulators of oncogenic rearrangement formation. Thus, the developed A549-based reporter provides a platform for future studies of cellular and environmental factors that influence chromosomal rearrangements and for further investigation of the underlying mechanisms.

## Data Availability

The original contributions presented in this study are included in the article/Supplementary Materials. Further inquiries can be directed to the corresponding author.

## Supplementary Materials

The following supporting information can be downloaded at: https://www.mdpi.com/article/10.3390/ijms27135676/s1.
