# Supplementary material for: ALK Knock-In Reporter Reveals APE1 as a Negative Regulator of EML4-ALK Formation
Source: Int J Mol Sci. 2026 Jun 24;27(13):5676. doi: 10.3390/ijms27135676 (PMC13362364; doi:10.3390/ijms27135676)
Supplement: Supplementary file 1 [file ijms-27-05676-s001.zip › ijms-4374342-supplementary.pdf]

Supplementary

Table S1. RT-qPCR primer sequences.

Gene name	Forward primer (5'-3')	Reverse primer (5'-3')
ACTB	ACTGGGACGACATGGAGAAA	GGCGTACAGGGATAGCACAG
EML4-ALK	AAGCCAAAATTTGTGCAGTGTT	TCGGTCATGATGGTCGAGGT
APEX1	CTGCTCTTGGAATGTGGATGGG	TCCAGGCAGCTCCTGAAGTTCA
BRCA2	GGCTTCAAAAAGCACTCCAGATG	GGATTCTGTATCTCTTGACGTTCC
XRCC5	GTTCTAAAGGTCTTTGCAGCAAGA	AAAAGCCACGCCGACTTGAGGA
RAD52	GCCCAGAATACATAAGTAGCCGC	CCACATTCTGCTGCGTGATGGA

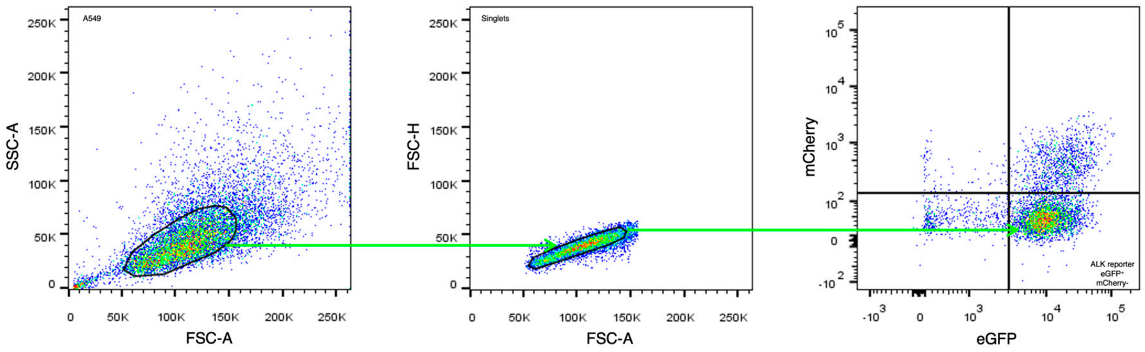

Figure S1. Gating strategy for sorting ALK reporter A549 cell line. Flow cytometry gating was performed sequentially by selecting cells on the basis of FSC-A/SSC-A, followed by FSC-H/FSC-A gating to select singlets. This population was then analyzed for eGFP and mCherry fluorescence, and the eGFP⁺/mCherry⁻ fraction was sorted as the reporter-positive population.

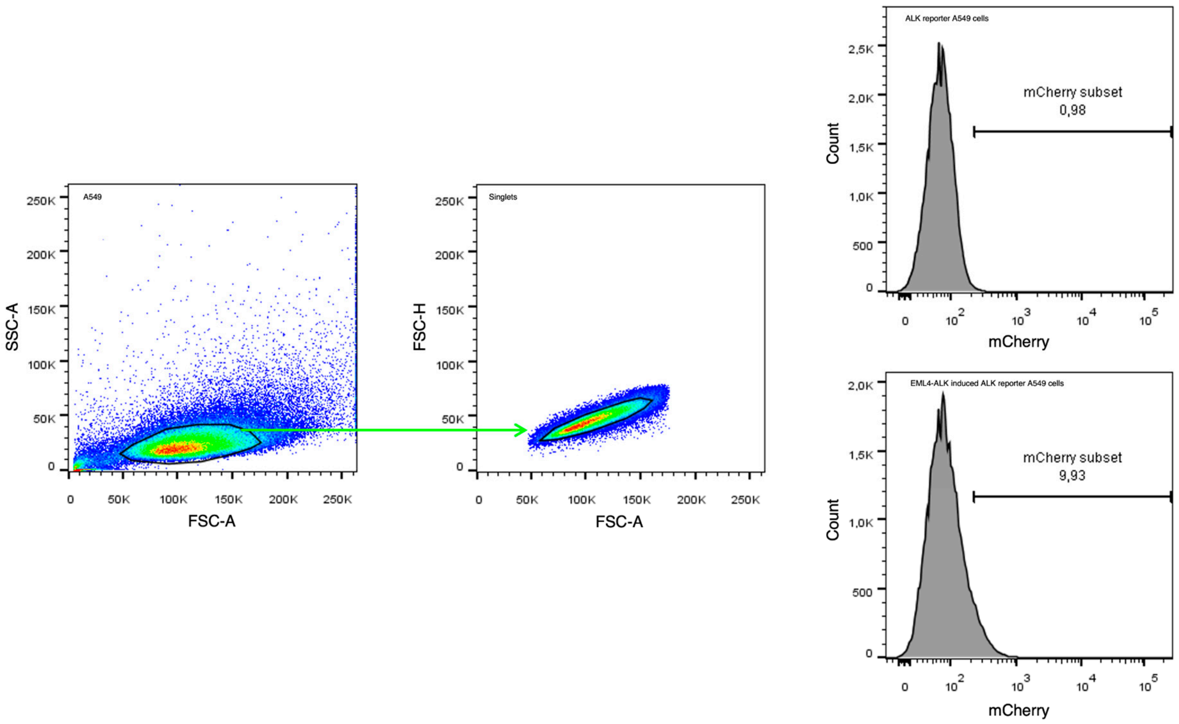

Figure S2. Gating strategy for mCherry-based quantification of reporter activation. Cells were gated by FSC-A/SSC-A and singlets were selected by FSC-H/FSC-A. A fixed mCherry threshold gate ("mCherry^{+} subset") was set on non-induced ALK reporter A549 cells to include ~1% of control cells and then applied unchanged to EML4-ALK-induced samples. Representative histograms are shown.

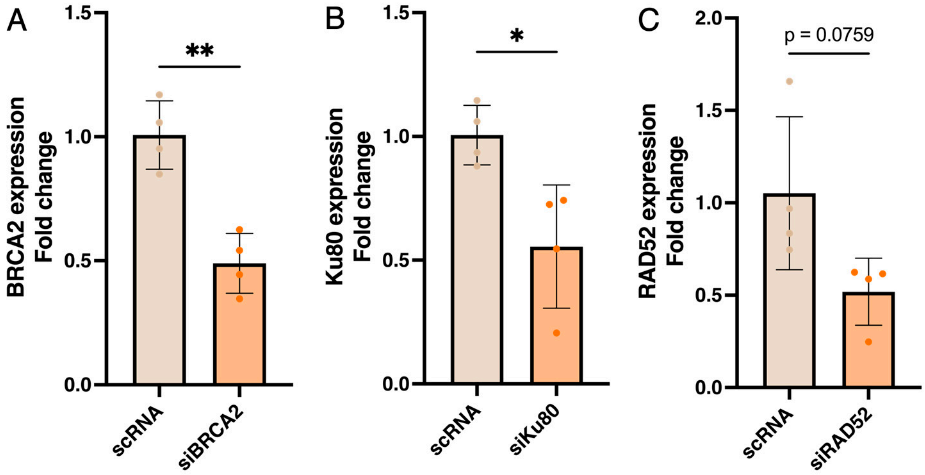

Figure S3. siRNA-mediated knockdown of DNA repair factors. Knockdown efficiencies of (A) *BRCA2*, (B) Ku80/*XRCC5*, and (C) *RAD52* mRNA in the ALK reporter A549 cell line were measured 7 days after siRNA transfection. Transcript levels were quantified relative to cells treated with non-targeting siRNA and normalized to *β-actin* using the $2^{-\Delta\Delta Ct}$ method. An unpaired *t*-test was used. The graph represents mean values ± SD from N = 4 biological replicates. Dots represent individual biological replicates. * – $p < 0.05$; ** – $p < 0.005$

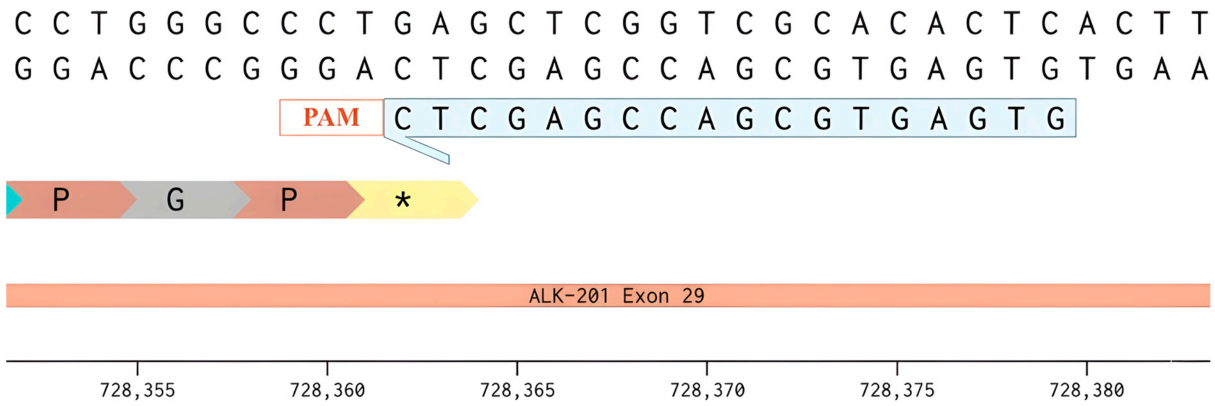

Figure S4. Design of the gRNA targeting the 3′ end of the ALK coding sequence. Genomic view of the *ALK* locus showing the crRNA selected for reporter knock-in. It was designed to span the junction between the stop codon and the 3′ untranslated region (3′ UTR), with the corresponding PAM partially overlapping the last coding codon in exon 29 of the *ALK-201* transcript. This design enables Cas9 cleavage in immediate proximity to the endogenous ALK stop codon, allowing precise insertion of the reporter cassette at the C terminus of ALK.

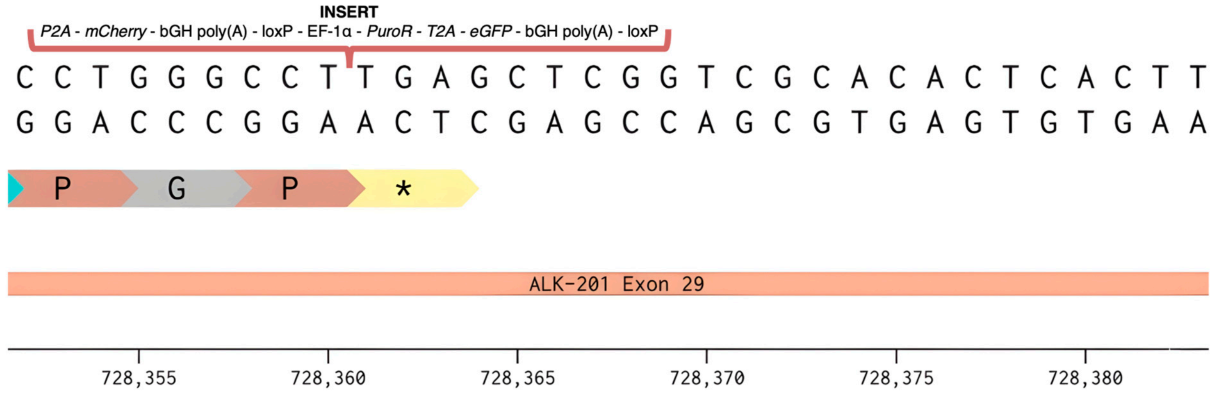

Figure S5. Reporter cassette insertion site at the 3′ end of the *ALK* gene. Genomic view of the knock-in site of the P2A-mCherry-EF1α-PuroR-T2A-eGFP cassette in the *ALK* gene. The insert was positioned immediately before the endogenous ALK stop codon to preserve expression from the native locus. A silent mutation was introduced into the last coding codon (CCC→CCT) without changing the encoded proline, providing an additional barrier to re-cutting.
